# Comparative Effectiveness of COVID-19 Vaccines in Preventing Infections and Disease Progression from SARS-CoV-2 Omicron BA.5 and BA.2, Portugal

**DOI:** 10.3201/eid2903.221367

**Published:** 2023-03

**Authors:** Irina Kislaya, Pedro Casaca, Vítor Borges, Carlos Sousa, Bibiana I. Ferreira, Ana Fonte, Eugénia Fernandes, Carlos Matias Dias, Sílvia Duarte, José Pedro Almeida, Inês Grenho, Luís Coelho, Rita Ferreira, Patrícia Pita Ferreira, Cláudia Medeiros Borges, Joana Isidro, Miguel Pinto, Luís Menezes, Daniel Sobral, Alexandra Nunes, Daniela Santos, António Maia Gonçalves, Luís Vieira, João Paulo Gomes, Pedro Pinto Leite, Baltazar Nunes, Ausenda Machado, André Peralta-Santos

**Affiliations:** Instituto Nacional de Saúde Doutor Ricardo Jorge, Lisbon, Portugal (I. Kislaya, V. Borges, C. Matias Dias, S. Duarte, L. Coelho, R. Ferreira, J. Isidro, M. Pinto, D. Sobral, A. Nunes, D. Santos, L. Vieira, J.P. Gomes, B. Nunes, A. Machado);; Comprehensive Health Research Centre, Lisbon (I. Kislaya, C. Matias Dias, B. Nunes, A. Machado, A. Peralta-Santos);; Direção-Geral da Saúde, Lisbon (P. Casaca, E. Fernandes, P. Pita Ferreira, P. Pinto Leite, A. Peralta-Santos);; Unilabs, Porto, Portugal (C. Sousa, J.P. Almeida, L. Menezes, A. Maia Gonçalves);; Algarve Biomedical Center Research Institute, Faro, Portugal (B.I. Ferreira, I. Grenho);; Administração Central do Sistema de Saúde, Lisbon (A. Fonte, C.M. Borges)

**Keywords:** COVID-19, coronavirus disease, SARS-CoV-2, severe acute respiratory syndrome coronavirus 2, viruses, respiratory infections, zoonoses, vaccine-preventable diseases, Omicron, BA.2, BA.5, postinfection vaccine effectiveness, hospitalization, death, Portugal

## Abstract

We estimated comparative primary and booster vaccine effectiveness (VE) of SARS-CoV-2 Omicron BA.5 and BA.2 lineages against infection and disease progression. During April–June 2022, we implemented a case–case and cohort study and classified lineages using whole-genome sequencing or spike gene target failure. For the case–case study, we estimated the adjusted odds ratios (aORs) of vaccination using a logistic regression. For the cohort study, we estimated VE against disease progression using a penalized logistic regression. We observed no reduced VE for primary (aOR 1.07 [95% CI 0.93–1.23]) or booster (aOR 0.96 [95% CI 0.84–1.09]) vaccination against BA.5 infection. Among BA.5 case-patients, booster VE against progression to hospitalization was lower than that among BA.2 case-patients (VE 77% [95% CI 49%–90%] vs. VE 93% [95% CI 86%–97%]). Although booster vaccination is less effective against BA.5 than against BA.2, it offers substantial protection against progression from BA.5 infection to severe disease.

The BA.5 lineage of the SARS-CoV-2 Omicron variant emerged in South Africa in February 2022 (*1*) and rapidly spread to other countries (*2*). In Portugal, the first BA.5 case was detected on March 29, 2022, and BA.5 became predominant by epidemiologic week 19 of 2022 (May 9–15) (*3*), leading to a new surge in SARS-CoV-2 infections, hospitalizations, and deaths (*4*).

Early data showed that protection against BA.4/5 conferred by a previous pre–Omicron SARS-CoV-2 variant infection was low (*5*). Studies from the United Kingdom (*6*) and Denmark (*7*) indicated no differences in the odds of having been vaccinated between BA.5 and BA.2 case-patients, suggesting no differences in the vaccine performance against infection. A severity assessment from South Africa indicated no differences in the risk for severe hospitalization or death during the BA.4/5 wave compared with the BA.1 wave (*8*). In contrast, a study from Denmark indicated higher odds of hospitalization among BA.5 case-patients compared with BA.2 case-patients, even among those vaccinated with a booster dose (*7*). Those studies have not addressed potential differences in vaccine effectiveness (VE) against severe outcomes or disease progression between the 2 lineages.

VE against emerging SARS-CoV-2 variants has become a pressing issue (*9*). In the context of highly vaccinated populations and challenges with establishing a negative control group, alternative study designs can be helpful. Case–case studies based on surveillance data that include only infected case-patients have been shown to be feasible for rapid evaluation of changes in VE against SARS-CoV-2 infection in the context of variants replacing other variants (*10*–*12*). Moreover, integrating the surveillance data on COVID-19 outcomes of different severity levels enables timely assessment of changes in VE against severe disease, which can be achieved by estimating VE against disease progression in infected case-patients (*13*,*14*). According to Halloran et al. (*14*), VE against severe outcomes estimated with traditional study designs can be expressed as a product of 2 components, VE against infection and VE against progression from infection to a severe outcome. Therefore, the reduction of VE against disease progression with a new variant would also lead to a decrease in VE against a severe outcome.

Our study builds on previous work to address this knowledge gap (*10*,*15*; A. Peralta-Santos, et al., unpub. data, https://doi.org/10.1101/2022.01.20.22269406). We aimed to measure the comparative VE of complete primary vaccination and first booster dose between Omicron BA.5 and BA.2 lineages against infection and compare lineage-specific VE against progression to severe COVID-19 outcomes that require hospitalization.

## Methods

### Study Design and Population

First, we conducted a case–case study to compare the odds of vaccination between persons infected with SARS-CoV-2 Omicron lineages BA.5 and BA.2. Second, we followed a cohort of BA.2- and BA.5-infected persons to compare lineage-specific VE against progression from infection to hospitalization (VEp). We estimated VEp by comparing the risk for severe outcomes in vaccinated infected and unvaccinated infected persons (*14*).

We included persons from mainland Portugal who had SARS-CoV-2 diagnosed by an reverse transcription PCR (RT-PCR) test and and had their illness reported to Portugal’s national surveillance system during April 25–June 10, 2022. We excluded SARS-CoV-2 case-patients who were not eligible for booster vaccination (i.e., those <18 years of age) and residents in the autonomous regions of Madeira and Azores or for whom residence information was unavailable. Further, we excluded those vaccinated with brands other than the ones used in Portugal; vaccinated with a combination of brands other than the ones recommended by the vaccines’ manufacturers; vaccinated with an interval period between the 2 doses shorter than that recommended by the manufacturer; vaccinated with the second booster dose or with an incomplete vaccination scheme; infected with variants other than BA.2 and BA.5 according to whole-genome sequencing (WGS) results. Finally, we excluded those who had suspected cases of nosocomial infection.

The testing policies remained stable during the study period, and all symptomatic patients were eligible for a free diagnostic test. All patients admitted to a hospital were tested at admission, even if asymptomatic. However, during April 29–May 23, 2022, rapid antigen tests were not available free of charge, and some asymptomatic infections might have been undiagnosed. The overall positivity rate during the study period was very high (≈50%) (*16*).

### Case Selection and Variant Classification

We classified samples as BA.2 or BA.5 according to spike gene target failure (SGTF) status (BA.5 as SGTF, BA.2 as non-SGTF) or by WGS. WGS data was provided by the National Genomics Surveillance Network, which conducts nationwide random sequencing surveys weekly (*3*). SGTF data was provided by 2 clinical pathology laboratories (UNILABS and Algarve Biomedical Center Laboratory) that operate in mainland Portugal and use the TaqPath COVID-19 RT-PCR (ThermoFisher, https://www.thermofisher.com), enabling identification of samples with SGTF or non-SGTF status. Those 2 laboratories detected ≈3% of diagnosed cases at the national level during the study period. For SGTF-based classification, we considered only samples with both nucleocapsid and open reading frame 1a positive signals and cycle threshold values <30.

We defined a COVID-19 hospitalization as any admission (of >24 hours’ duration) of a patient to the National Health Service (NHS) hospitals in mainland Portugal with a SARS-CoV-2 diagnosis classified as BA.2 or BA.5 infection. We obtained data from Portgual’s Integrated Hospital Information System registry, which captures information from NHS hospitals and registers COVID-19 admissions for all the patients with primary or secondary COVID-19 diagnoses hospitalized in COVID-19 dedicated facilities. In Portugal, NHS covers the cost of nearly all COVID-19 hospitalizations.

### Exposure Definition

We extracted vaccination status from the nationwide electronic vaccination registry and classified them as unvaccinated (i.e., no record of COVID-19 vaccine administration), complete primary vaccination received, or booster dose vaccination received. We included a patient in the primary vaccination category if the SARS-CoV-2 infection diagnosis occurred >14 days after the complete vaccination regimen according to the product characteristics (i.e., >14 days after the second dose of mRNA BNT162b2 [Comirnaty, https://www.pfizer.com], mRNA-1273 SARS-CoV-2 [Moderna, https://www.modernatx.com] or AstraZeneca [https://www.astrazeneca.com] vaccines or >14 days after the single dose of the Johnson & Johnson/Janssen [https://www.jnj.com] COVID-19 vaccine). We included a patient in the booster dose vaccination if a SARS-CoV-2 infection diagnosis occurred >14 days after the first mRNA booster dose.

### Other Covariates

We collected information on age, sex, region of residence, and swab collection date through Portugal’s national surveillance system. We defined a previous infection as a positive RT-PCR or rapid antigen SARS-CoV-2 test result for the same patient >90 days apart. Data extraction and deterministic linkage of electronic health records with laboratory data were performed on July 12, 2022, by the General Directorate of Health team using the National Health Service user number, a unique identifier for health services in Portugal.

### Statistical Analysis

We used absolute and relative frequencies to describe BA.2 and BA.5 case characteristics. In a case–case design, we estimated the odds of vaccination (primary and first booster dose) and previous infection in BA.5 case-patients compared with BA.2 case-patients by using a logistic regression model adjusted for sex, age group, region of residence, and week of swab collection.

For interpretation, we expect no differences in VE between 2 lineages if the odds of vaccination in BA.5 case-patients are higher than for BA.2 case-patients, the OR estimate is >1, suggesting that VE is lower for BA.5 lineage compared with BA.2. If the odds of vaccination are similar between BA.2 and BA.5, (i.e., OR = 1), we expect no differences in VE between 2 lineages. The OR for the previous infection can be interpreted similarly; an OR >1 suggests less protection conferred by the previous infection against BA.5 compared with BA.2. In addition, we combined previous infection and vaccination exposure to compare levels of protection conferred by so-called hybrid immunity between BA.5 and BA.2 lineages.

To reduce the bias caused by rare events, we estimated VEp in BA.5 and BA.2 case-patients by using penalized logistic regression (Firth’s penalized likelihood method) (*17*), adjusting for sex, age group, region of residency, and week of swab collection. To compare lineage-specific VEp estimates, we included an interaction term between lineage and vaccination status in the models. The OR for the lineage and vaccination status interaction can be interpreted as measure of relative VE to prevent progression to severe outcomes among patients infected with BA.5 compared with BA.2.

We performed all statistical analyses with Stata 15.1 software (StataCorp, https://www.stata.com). All tests were 2-sided, and we considered a p value <0.05 to be statistically significant.

### Ethics Considerations

The genomic surveillance of SARS-CoV-2 in Portugal is regulated by the Assistant Secretary of State and Health Executive Order (dispatch no. 331/2021, issued January 11, 2021). The study protocol received clearance from the Ethics Committee of Portugal’s Instituto Nacional de Saúde Doutor Ricardo Jorge on June 15, 2022.

## Results

### Study Participant Characteristics

For the period April 25–June 10, 2022, we included 27,702 SARS-CoV-2–positive case-patients (15,396 with the BA.2 variant and 12,306 with BA.5). A total of 106 COVID-19 hospitalization occurred (54 [0.4%] among patients infected with BA.2 and 52 [0.4%] among those infected with BA.5). Most cases (91.2%) were classified using SGTF. Sex distribution was similar between the 2 groups (BA.5 case-patients were slightly younger than BA.2 case-patients), and BA.5 was more frequent in Alentejo and Centro regions (Table 1). Both groups had a similar percentage of nonvaccinated case-patients (4%–5%), but the BA.5 group had a higher percentage of case-patients who received complete primary vaccination (20.6% vs. 15.8%), and BA.2 case-patients had a higher percentage of patients who had received the first booster dose (80.1% vs. 74.7%). Also, the percentage of case-patients with a previous COVID-19 infection was higher among BA.5 case-patients (10.0%) than among BA.2 case-patients (5.6%).

**Table 1:** Sociodemographic and clinical characteristics of COVID-19 case-patients in the study sample, by SARS-CoV-2 variant, Portugal, April 25–June 10, 2022

Characteristic	No. (%) patients			
	BA.5, n = 12,306		BA.2, n = 15,396	
Sex				
F	7,176 (58.3)		9,043 (58.7)	
M	5,130 (41.7)		6,353 (41.3)	
Age group, y				
18–29	3,474 (28.2)		3,299 (21.4)	
30–39	2,059 (16.7)		2,922 (19.0)	
40–49	2,475 (20.1)		3,431 (22.3)	
50–59	1,974 (16.0)		2,581 (16.8)	
60–69	1,089 (8.9)		1,567 (10.2)	
>70	1,235 (10.0)		1,596 (10.4)	
Region				
Alentejo	1,280 (10.4)		752 (4.9)	
Algarve	325 (2.6)		487 (3.2)	
Centro	1,401 (11.4)		972 (6.3)	
Lisboa e Vale do Tejo	1,462 (11.9)		3,396 (22.1)	
Norte	7,838 (63.7)		9,789 (63.6)	
Epidemiologic week of diagnosis, 2022				
Week 17	980 (8.0)		4,200 (27.3)	
Week 18	3,237 (26.3)		5,691 (37.0)	
Week 19	5,655 (46.0)		4,763 (30.9)	
Week 20	1,080 (8.8)		492 (3.2)	
Week 21	799 (6.5)		174 (1.1)	
Week 22	348 (2.8)		56 (0.4)	
Week 23	207 (1.7)		20 (0.1)	
COVID-19 vaccination status				
Not vaccinated	590 (4.8)		631 (4.1)	
Complete primary vaccination	2,530 (20.6)		2,434 (15.8)	
First booster vaccination	9,186 (74.7)		12,331 (80.1)	
Previous SARS-CoV-2 infection				
No	11,073 (90.0)		14,536 (94.4)	
Yes	1,233 (10.0)		860 (5.6)	
Hospitalization				
No	12,254 (99.6)		15,342 (99.7)	
Yes	52 (0.4)		54 (0.4)	

### Case–Case Study

For the case–case study, the odds of complete primary vaccination (aOR 1.07 [95% CI 0.93–1.23]) or first booster dose (aOR 0.96 [95% CI 0.84–1.09]) among BA.5 case-patients were similar to those for the BA.2 case-patients, suggesting no relevant differences in VE against infection for the BA.5 lineage compared with the BA.2 lineage (Table 2). We observed higher odds of previous infection in BA.5 case-patients compared with BA.2 case-patients (aOR 1.44 [95% CI 1.30–1.60]).

**Table 2:** Crude and adjusted odds ratios of vaccine infection breakthrough in BA.5 case-patients compared with BA.2 SARS-CoV-2 case-patients, Portugal, epidemiologic weeks 17–23, 2022*

Category	BA.5, no. (%)	BA.2, no. (%)	Crude OR (95% CI)	Adjusted† OR (95% CI)
Vaccination status				
Unvaccinated	590	631	Referent	Referent
Complete primary vaccination	2,530	2,434	1.11 (0.98–1.26)	1.07 (0.93–1.23)
Booster dose	9,186	12,331	0.80 (0.71–0.89)	0.96 (0.84–1.09)
Previous infection				
No	11,073	14,536	Referent	Referent
Yes	1,233	860	1.88 (0.71–2.06)	1.44 (1.30–1.60)
Vaccination status accounting for previous infection				
Unvaccinated without previous infection	468 (3.8)	550 (3.6)	Referent	Referent
Unvaccinated with previous infection	122 (1.0)	81 (0.5)	1.77 (1.30–2.41)	1.77 (1.26–2.49)
Complete primary vaccination without previous infection	1,802 (14.6)	1,982 (12.9)	1.07 (0.93–1.23)	1.08 (0.92–1.26)
Complete primary vaccination with previous infection	729 (5.9)	452 (2.9)	1.90 (1.60–2.25)	1.70 (1.40–2.05)
Booster without previous infection	8,805 (71.5)	12,004 (78.0)	0.86 (0.76–0.98)	0.99 (0.86–1.14)
Booster with the previous infection	382 (3.1)	327 (2.1)	1.37 (1.13–1.66)	1.18 (0.95–1.47)

*OR, odds ratio. †Adjusted for age group, sex, region, and week of diagnosis.

### Cohort VEp Study

For the cohort study, regarding hospitalization (Table 3), for complete primary vaccination, we estimated an aOR of 0.38 (95% CI 0.16–0.89) for BA.2 case-patients and 0.78 (95% CI 0.29–2.09) for BA.5 case-patients, which is equivalent to a VEp of 62% (95% CI 11%–84%) for BA.5 and 22% (95% CI –109%–71%) for BA.2 For the first booster dose, we observed a higher reduction in risk for hospitalization among infected patients for both BA.2 (aOR 0.07 [95% CI 0.03–0.14]) and BA.5 (aOR 0.23 [(95% CI 0.10–0.51]), representing VEp of 93% (95% CI 86%–97%) for BA.2 and 77% (95% CI 49%–90%) for BA.5.

**Table 3:** Adjusted odds ratios of hospitalization among COVID-19 case-patients, by SARS-CoV-2 variant, Portugal, 2022*

Vaccination status	BA.5			BA.2		aOR BA.5/BA.2 (95% CI)
	No. (%)	aOR (95% CI)		No. (%)	aOR (95% CI)	
Not vaccinated	9/590 (1.53)	Referent		14/631 (2.2)	Referent	
Complete primary vaccination	9/2,530 (0.36)	0.78 (0.29–2.09)		11/2,434 (0.45)	0.38 (0.16–0.89)	2.06 (0.56–7.55)
1st booster vaccination	34/9,186 (0.37)	0.23 (0.10–0.51)		29/12,331 (0.24)	0.07 (0.03–0.14)	3.36 (1.18–9.63)

*aOR, adjusted odds ratio; OR, odds ratio.

The interaction term that enables comparison between BA.5 and BA.2 lineage-specific VEp was aOR 3.36 (95% CI 1.18–9.63), suggesting reduced protection induced by the first booster dose against hospitalization for BA.5 case-patients compared with BA.2. For complete primary vaccination the difference in VEp between BA.5 and BA.2 case-patients was not statistically significant (aOR 2.06 [95% CI 0.56–7.55]).

## Discussion

Using routinely collected data from electronic health records, we found no differences in odds of vaccination between BA.5 and BA.2 infection in the adult population of Portugal, suggesting that VE against BA.5 infection was similar to VE against BA.2. This result corroborates findings from studies conducted in the United Kingdom and Denmark that compared VE against infection between BA.5 and BA.2 by using a similar methods (*6*,*7*).

Our study showed that infection with the SARS-CoV-2 Omicron BA.5 lineage was associated with higher odds of previous infection compared with BA.2, suggesting reduced protection conferred by the previous infection against BA.5. The effect of the previous infection on the odds of being infected with BA.4/BA.5 and BA.2 has been investigated in Qatar, where the reported protective effect of previous infection against infection with BA.2 was 46.1% (95% CI 39.5%–51.9%) (*18*). The authors reported a low effect for pre-Omicron infection 14.9% (95% CI −47.5%–50.9%) and higher effectiveness for previous infection with BA.1/2 of 76.1% (95% CI 54.9%–87.3%) in reducing the risk for infection with BA.4/BA.5 (*5*). Although not directly comparable, our results align with these findings.

Moreover, we used a cohort design to compare the risk for hospitalization among vaccinated and unvaccinated patients, conditional on being infected with BA.5 or BA.2. Our results suggest statistically significant differences between BA.5 and BA.2 in VEp after the first booster dose (aOR 3.36 [95% CI 1.18–9.63]). In addition, among BA.5-infected patients, the protective effect of the first booster on reducing the odds of hospitalization was higher (VEp 77% [95% CI 49%–90%]) than for the primary vaccination (VEp 22%–95% [95% CI −109% to 71%]). These findings align with neutralization studies that suggested higher immune evasion for the BA.5 lineage than for BA.2 (*19*) and an improvement in plasma-neutralizing activity after receipt of booster vaccine, highlighting the importance of vaccine boosters for eliciting potent neutralizing antibody responses against Omicron lineages (*20*).

Among the limitations of our study is that, for most included cases, SARS-CoV-2 variant was determined by SGTF, so we cannot exclude the possibility of variant misclassification, given that other contemporary lineages (BA.1 and BA.4) also display SGTF and non-SGTF status. However, genomic surveillance data indicate that this potential bias was largely minimized because BA.1 and BA.4 had a <0.3% weekly relative frequency throughout the study period (*3*). Regarding the non-SGTF profile, only very sporadic sequences were detected beside the dominant BA.2. Both observations support a reduced risk for lineage misclassification.

In addition, the study relies on surveillance data that had some limitations (e.g., lack of information on potential confounders, underlying conditions, and adherence to protective measures such as mask use, social distancing, or other behaviors, which may differ between vaccinated and unvaccinated patients). These differences can be rooted in the risk perception of the disease associated with age or previous exposure to SARS-CoV-2. Although we account for age, sex, and region of residence in the models, which minimize the confounding, we cannot exclude unmeasured confounding bias.

Moreover, we cannot identify the variant from a previous infection, and having a pre-Omicron infection affects the odds of being infected with BA.5, as demonstrated in the Qatar study (*5*,*18*). Time since previous infection was also unknown, so we were not able to address the hypothesis of waning protection. We did not account for the underascertainment of the previous infection, meaning that we are probably underestimating the protective effect of the previous infection. Serologic surveys have estimated postinfection seroprevalence to be higher than the cumulative incidence reported by the national surveillance system in Portugal (*21*,*22*).

The ascertainment bias might be present if the probability of testing is different between vaccinated and unvaccinated case-patients. However, during the study period, the daily testing rate in Portugal was 3.6–4.7 tests/1,000 population, the testing recommendations remained stable, and active community testing was maintained, which meant all symptomatic persons could have a free test and paid sick leave regardless of vaccination status. The variant status is unknown to the person being tested and hence is less likely to be an incentive for different testing behaviors by itself. Although we cannot exclude the effect of ascertainment bias on our results, the robust community testing and the paid sick leave program probably minimize it.

Our approach does not provide a direct measure of VE against infection or severe disease but does provide a rapid assessment of the effects of SARS-CoV-2 variants on VE, which can be helpful in guiding public health measures. Our results suggest no differences in VE against SARS-CoV infection and lower protective effect of previous infection against infection with BA.5 compared with BA.2, which explains the surge in cases observed in countries with high BA.5 prevalence. In addition, we observed that VE against COVID-19 progression to severe disease was lower among patients infected by BA.5 compared with BA.2. Vaccines currently used in Portugal are less effective in reducing the risk for disease progression to severe outcomes for patients infected with BA.5 compared with BA.2. The observed difference between BA.5 and BA.2 lineages emphasizes the importance of high vaccination coverage to prevent severe COVID-19–associated outcomes.
